# Effects of prenatal subjective well-being on birth outcomes and child development: A longitudinal study

**DOI:** 10.1192/j.eurpsy.2022.2338

**Published:** 2022-11-04

**Authors:** Wanda Estinfort, Jian-Pei Huang, Heng-Kien Au, Chen-Li Lin, Yi-Yung Chen, Hsing Jasmine Chao, Ling-Chu Chien, Yu-Chun Lo, Yi-Hua Chen

**Affiliations:** 1 School of Public Health, College of Public Health, Taipei Medical University, Taipei, Taiwan; 2 Department of Obstetrics and Gynecology, Mackay Memorial Hospital, Taipei, Taiwan; 3 Department of Obstetrics and Gynecology, Taipei Medical University Hospital, Taipei, Taiwan; 4 Department of Obstetrics and Gynecology, School of Medicine, College of Medicine, Taipei Medical University, Taipei, Taiwan; 5 Department of Obstetrics and Gynecology, Taipei City Hospital, Heping Fuyou Branch, Taipei, Taiwan; 6 Neuroscience Research Center, Taipei Medical University, Taipei, Taiwan; 7 Neural Regenerative Medicine, College of Medical Science and Technology, Taipei Medical University, Taipei, Taiwan; 8 Research Center of Health Equity, College of Public Health, Taipei Medical University, Taipei, Taiwan

**Keywords:** Subjective well-being, birth outcomes, early development, parity

## Abstract

**Background:**

Although maternal mental illnesses have been found to influence child health and development, little is known about the impact of maternal positive well-being on child health and development. Therefore, this longitudinal study investigated the effects of prenatal subjective well-being on birth outcomes and child development by considering the potential modifier effect of parity.

**Methods:**

Pregnant women in early stages of pregnancy were recruited at five selected hospitals in Taipei, Taiwan, during their prenatal appointments since 2011. Self-reported evaluations were conducted at seven time points up to 2 years postpartum. Linear regression and generalized estimating equation models were used for examination.

**Results:**

Higher prenatal eudaimonic well-being was associated with longer gestational length (adjusted beta [aβ] = 0.36, 95% confidence interval [CI] = 0.03, 0.68) and higher birth weight (aβ = 124.71, 95% CI = 35.75, 213.66). Higher positive and negative affect were associated with longer gestational length (aβ = 0.38, 95% CI = 0.06, 0.70) and smaller birth weight (aβ = −93.51, 95% CI = −178.35, −8.67), respectively. For child’s outcomes, we found an association between higher prenatal eudaimonic well-being and decreased risks of suspected developmental delay, particularly for children of multiparous mothers (adjusted odds ratio = 0.18, 95% CI = 0.05, 0.70). Higher levels of prenatal depression and anxiety were significantly associated with increased risks of suspected developmental delay for children of primiparous mothers.

**Conclusions:**

Positive prenatal maternal mental health may benefit birth outcomes and child development, particularly for children of multiparous mothers. Interventions for improving prenatal mental health may be beneficial for child development.

## Introduction

Subjective well-being (SWB) is a multidimensional measure that involves cognitive (life satisfaction and positive functioning) and emotional components subjective to an individual and relevant to society’s norms and values [1]. The European Commission and the Organization for Economic Cooperation and Development (OECD) guidelines are commonly adopted to define SWB, which encompasses three fundamental components: life evaluation, eudaimonic well-being (i.e., a sense of purpose and meaning in life), and affect (positive and negative) [2]. SWB has long been considered critical to positively affect several health outcomes including decreased mortality, decreased cardiovascular disease risks, fewer chronic illnesses and functional impairments, and improved health behavior [3, 4]. Extending the examination of well-being from pregnancy to postpartum years is crucial to maximizing wellness of maternal and child health [5].

Previous studies on parental happiness trajectories found that parental happiness increased in the year before delivery and then fell to pre-child levels [6]. Parental happiness has also been observed to be influenced by factors such as parity, with parents who had a second child having a greater baseline level of life satisfaction than those who only had one child [6]. Although accumulated evidence indicated that poor perinatal maternal mental health might have a negative impact on the child physically [7], psychologically [8, 9], emotionally, and behaviorally [10], few studies have investigated the effects of maternal positive well-being on child outcomes.

Regarding birth outcomes, a longitudinal study reported that positive affect in the first and second trimesters was associated with longer gestational length, and higher positive emotion in late pregnancy was associated with a lower risk of preterm delivery [11]. For child development, on the other hand, parental psychological well-being is associated with health behavior [12] and parenting behavior [13], which may impact child development [14]. For example, prenatal maternal positive mental health was associated with a lower risk of mental illness in children [15] and reduced the effect of maternal stress on children internalizing and externalizing symptoms [16]. In addition, a longitudinal study reported that positive maternal perinatal mental health is associated with more favorable developmental outcomes in toddlers, particularly regarding cognition, communication, and social development [17]. To the best of our knowledge, no study has broadly evaluated the fundamental components of maternal SWB simultaneously. Moreover, the association between maternal positive well-being and child development has been rarely investigated, and the moderating effect of parity has not been addressed.

Our study addresses previous research limitations by using a longitudinal design to assess SWB from early pregnancy to 2 years postpartum; as well as the negative effects of depression and anxiety, separately, on birth outcomes and child development, with the potential moderating effects of parity examined. We hypothesize that the children of mothers with higher levels of SWB would be less likely to have a suspected developmental delay than the children of mothers with lower levels of SWB and that the effect could differ between first-time and experienced mothers.

## Methods

### Study design and sample

We analyzed data from the Longitudinal Examination across Prenatal and Postpartum Health in Taiwan (LEAPP-HIT) project, which is an ongoing prospective study that began in 2011 in Taipei, Taiwan. Pregnant women were invited to participate in the study during prenatal visits at outpatient clinics of five selected hospitals; they were then followed by postal delivery of surveys coupled with phone reminders to increase the response rate. We recruited women aged 20 years and over, whose pregnancies had not exceeded 16 gestational weeks, who intended to bring the baby to term, who were proficient in Chinese, and whose partners were willing to participate in the study. Those with physical or mental difficulties and with multigestational pregnancies (*n* = 10) were excluded. Detailed information on the cohort has been published elsewhere [18].

Self-reported instruments were used to assess the most important early developmental years. Overall, assessments were conducted at seven time points: early pregnancy (prior to 16 weeks, T1), mid-pregnancy (17 to 28 weeks, T2), late pregnancy (after 29 weeks, T3), and 1 month (T4), 6 months (T5), 1 year (T6), and 2 years (T7) postpartum. A total of 1,224 participants completed the questionnaires from T1 to T4 (up to childbirth), and 600 completed from T1 to T7 (up to 2 years postpartum). Some participants for whom we had data up to birth outcomes had not yet reached the 2-year mark to compare the differences because our cohort is part of an ongoing project. In addition, we only added the SWB instrument in 2016; therefore, we analyzed the positive mental health of 454 women. No significant differences were observed regarding sociodemographic factors and maternal emotional disturbance for women with and without SWB assessments. Written informed consent was obtained from each participant. The study protocol was approved by the institutional review boards of the participating hospitals.

## Instruments and Measures

In general, maternal SWB, depression, and anxiety were assessed from T1 to T7, while the child’s development from T5 to T7 was examined. In addition, we further derived sociodemographic data at T1 and child characteristics (e.g., birth outcomes, sex, and birth order) at T4 from the questionnaires. Detailed descriptions are presented later in the article.

### Outcome variables

#### Birth outcomes and child development

Children’s gestational weeks and birth weight were reported by mothers at T4 based on the records in the “Children’s Health Booklet,” which were assessed by hospital healthcare professionals. Gestational weeks and birth weight were used as continuous variables.

We used the Taipei City Developmental Checklist for Preschoolers, 2nd Edition (Taipei II) [19] to assess suspected developmental delay in children aged from 6–24 months (T5 to T7). This concise screening instrument is designed to identify children that should be referred for further evaluation due to a potential risk of developmental delay. It contains 13 checklists for 13 age groups, with each consisting of 11–13 behavior- or skill-related items relating to gross and fine motor control, cognition, language/communication, and emotional/social areas. The items were easily observable or elicited by the child’s caregiver. The internal consistency coefficients (α) of the 13 checklists were 0.72–0.87. A child was classified to have “suspected developmental delay” if they failed more than two items or had more than one marked item throughout the evaluation. This cutoff point had a sensitivity of 0.8 and a specificity of 0.99 and helped to evaluate possible delays in children aged less than 3 years [19].

### Independent variables

#### Subjective well-being

The guidelines of the Joint Research Centre of the European Commission and the OECD were used to assess elements of SWB from T1 to T7 [2]. Four items were used for life evaluation, with questions including “I am satisfied with my life,” and six items were used to measure eudaimonic well-being, with questions including “Most days I get a sense of accomplishment from what I do.” Six items were used for evaluating affect, three for positive (e.g., happy) and three for negative affect (e.g., sad) [2, 20]. Responses were rated on a five-point Likert scale (from 5-strongly agree to 1-strongly disagree). Appropriate validity and reliability have been reported for the scales of life evaluation [21, 22], eudaimonic well-being, and positive and negative affect [23]. Although continuous scores were often preferred in statistical modeling because they provided greater variability, we also sought to identify women with psychological issues below the regular line. To assess prenatal effects on birth outcomes using linear regression models, the median was used to dichotomize the average of T2 and T3 assessments to indicate higher or lower levels of each SWB throughout pregnancy. Whereas for developmental outcomes using the generalized estimating equations models (GEE) to emphasize the trajectories, the median score at each time point from T4 to T7 was used as the cutoff to dichotomize elements of SWB during the postpartum period.

#### Depression and anxiety

The Edinburgh Postnatal Depression Scale (EPDS) [24] was used to measure depression. Adequate reliability and validity have been demonstrated for the EPDS for Chinese mothers [25]. We used 20 items from the State subscale of the State–Trait Anxiety Inventory (STAI) [26] to assess anxiety. The Chinese version of the STAI has been validated with high reliability [27].

We used a cutoff value to address higher or lower levels of depression and anxiety (i.e., 14/13 for depression [28] and 45/44 for anxiety [29]) to dichotomize the average score of T2 and T3 and assess the effects on birth outcomes. In addition, the same cutoff value was used at each postnatal time point from T4 to T7 in the GEE model to emphasize the trajectories’ effects on child developmental outcomes.

#### Covariates

At T1, participants completed the baseline questionnaire that collected information on age, education level, employment status, household income, unplanned pregnancy, smoking status, drinking status, perceived health status, and parity. We also collected information on the child’s birth sex (T4) and health status from birth to 2 years old (T4 to T7).

### Statistical analysis

Participant characteristics were summarized using descriptive statistics. Changes in maternal SWB scores over time (from T1 to T7) were examined using repeated-measures analysis of variance (ANOVA). We conducted group-based trajectory modeling (GBTM) to collect individuals who had statistically similar trajectories into meaningful subgroups of prenatal mental health (T1 to T3). We used GBTM to ensure that we thoroughly used the longitudinal data during the prenatal period and identified higher-risk women requiring special attention. Grouping from GBTM was used in the GEE to examine the effects of prenatal mental health throughout the child’s development. To accurately define the trajectories, the relationship between time (i.e., pregnancy trimester) and outcome (e.g., life evaluation and depression) was modeled using polynomial equations. The optimal model, in terms of the number and shape of trajectories, was then chosen using the Bayesian Information Criterion, which favored parsimony [30]. Stata plugins traj were used for the analyses [30].

Associations between prenatal positive and negative mental health and birth outcomes were examined using linear regression models. GEEs with an exchangeable correlation structure were used to investigate the effects of maternal positive and negative mental health trajectories on child developmental outcomes from T5 to T7. Each mental health domain was examined in separate models, with time-invariant variables (e.g., prenatal mental health from GBTM) and time-varying variables (e.g., postnatal mental health from T4 to T7) simultaneously. Previously proposed potential confounders or variables with (*p* < 0.2) in univariate analyses were included in the multivariable regression model. Interaction terms between parity and the main independent variables reached statistical significance (*p* < 0.1) for child development outcomes but not for birth outcomes (*p*-values 0.3–0.7); therefore, subgroup analyses stratified by parity were further performed to model child development. Data were analyzed using the Stata software program (Release 15; Stata Corp LLC, College Station, TX). Statistical significance was set at *p* < 0.05 (two-tailed).

## Results

### GBTM selection and evaluation

The GBTM results indicated that a two-group solution met the criteria for adequate model fit. Among the two-trajectories groups, over 60% of the participants were classified to have high life evaluation, eudaimonic well-being, and positive affect trajectories, whereas 29.0, 34.1, and 31.2% of the participants were classified to have a high negative affect, depression, and anxiety, respectively.

Table 1 presents the distribution of prenatal life evaluation and eudaimonic well-being according to participant characteristics (see the Supplementary Materials for affect, depression, and anxiety).

**Table 1.** Distribution of prenatal life evaluation and eudaimonic well-being by demographics and maternal and infant characteristics.

	Life evaluation			Eudaimonic well-being		
	Lower	Higher		Lower	Higher	*p*-value
Variables	*n* (%)	*n* (%)	*p*-value	*n* (%)	*n* (%)	
Maternal age						
<35	102 (37.1)	173 (62.9)		91 (33.1)	184 (66.9)	
≥35	63 (36.0)	112 (64.0)	0.81	62 (35.4)	113 (64.6)	0.61
Education level						
High school or below	7 (29.2)	17 (70.8)		5 (20.8)	19 (79.2)	
Undergraduate and above	160 (37.4)	268 (62.6)	0.42	149 (34.8)	279 (65.2)	0.16
Employment status						
Yes	139 (36.0)	247 (64.0)		123 (32.0)	263 (68.0)	
No	27 (40.9)	39 (59.1)	0.45	30 (45.4)	36 (54.6)	0.03*
Monthly family income						
Less than NT$ 60,000	38 (52.0)	35 (48.0)		37 (50.7)	36 (49.3)	
NT$ 60,000–100,000	84 (38.5)	134 (61.5)	0.001**	77 (35.3)	141 (64.7)	<0.001***
More than NT$ 100,000	44 (27.2)	118 (72.8)		39 (24.1)	123 (75.9)	
Smoking						
Yes	7 (63.6)	4 (36.4)	0.11	8 (72.7)	3 (27.3)	0.01*
No	158 (36.7)	263 (63.3)		143 (33.2)	288 (66.8)	
Drinking						
Yes	6 (35.3)	11 (64.7)		8 (47.1)	9 (52.9)	
No	160 (36.8)	275 (63.2)	0.90	46 (33.6)	289 (66.4)	0.23
Mother main care giver						
Yes	152 (37.7)	251 (62.3)		137 (34.0)	266 (66.0)	
No	15 (30.6)	34 (69.4)	0.33	17 (34.7)	32 (65.3)	0.92
Depression						
Higher	95 (61.7)	59 (38.3)		97 (63.0)	57 (37.0)	
Lower	64 (22.5)	221 (77.5)	<0.001***	49 (17.2)	236 (82.8)	<0.001***
Anxiety						
Higher	79 (64.2)	44 (35.8)		80 (65.0)	43 (35.0)	
Lower	80 (25.3)	236 (74.7)	<0.001***	66 (20.9)	250 (79.1)	<0.001***
Parity						
Primiparous	107 (35.5)	194 (64.5)		100 (33.2)	201 (66.8)	
Multiparous	60 (39.2)	93 (60.8)	0.44	54 (35.3)	99 (64.7)	0.66
Mother perceived health						
Very good	1 (2.6)	37 (97.4)		2 (5.3)	36 (94.7)	
Good	138 (36.3)	242 (63.7)	<0.001***	131 (34.5)	249 (65.5)	<0.001***
Bad	27 (77.1)	8 (22.9)		21 (60.0)	14 (40.0)	
Fetus health problem						
Yes	70 (36.5)	122 (63.5)		72 (37.5)	120 (62.5)	
No	96 (36.8)	165 (63.2)	0.94	82 (31.4)	179 (68.6)	0.18
Infant’s sex						
Boy	84 (37.5)	140 (62.5)		72 (32.1)	152 (67.9)	
Girl	77 (35.0)	143 (65.0)	0.58	77 (35.0)	143 (65.0)	0.52
Planned pregnancy						
Yes	61 (44.8)	75 (55.2)		45 (33.1)	91 (66.9)	
No	103 (32.8)	211 (67.2)	0.02*	106 (33.8)	208 (66.2)	0.89

*Note*: The total count for each variable may vary because of missing values.

*p < 0.05, **p < 0.01, ***p < 0.001.

### Repeated measure ANOVA results

**Figure 1.**
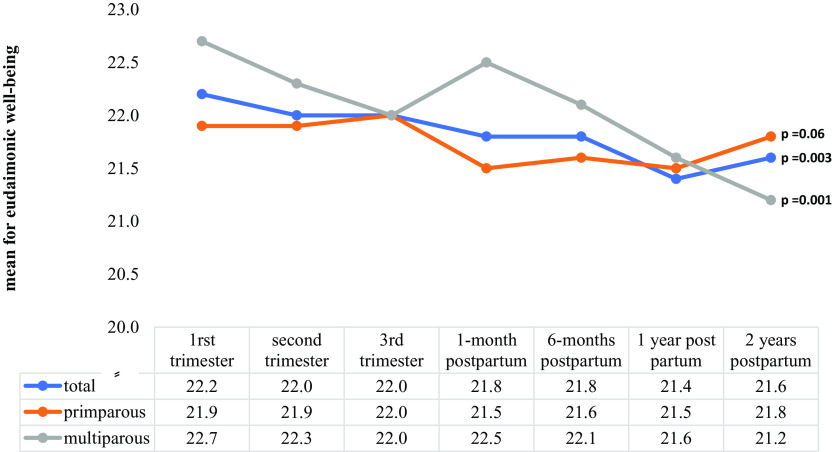
Changes in maternal eudaimonic well-being scores from early pregnancy to 2 years postpartum by parity.

**Figure 2.**
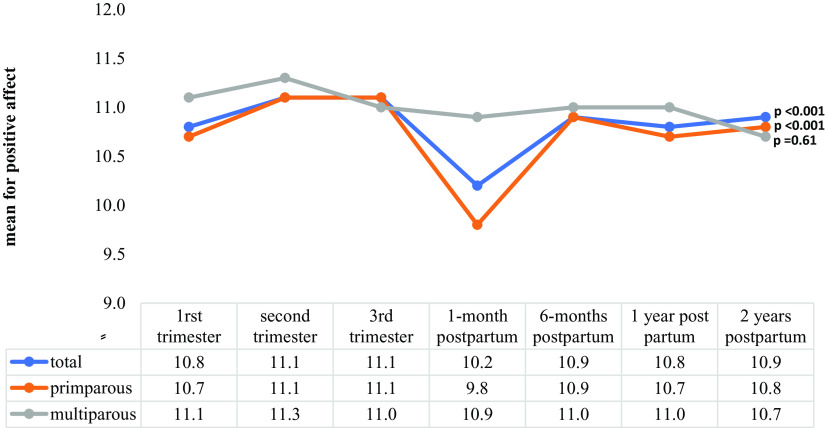
Changes in maternal positive affect scores from early pregnancy to 2 years postpartum by parity.

Repeated-measures ANOVA scores for maternal eudaimonic well-being (*p* = 0.003) and positive affect (*p* < 0.001) exhibited significant changes from T1 to T7 (Figures 1 and 2). Significant changes in eudaimonia scores were noted for multiparous mothers (*p* = 0.001; Figure 1), and significant changes in maternal positive affect scores were observed for primiparous mothers (*p* < 0.001) from T1 to T7.

Table 2 presents the associations between prenatal positive and negative mental health and birth outcomes, after adjustment for relevant covariates. The results indicated that higher prenatal eudaimonic well-being was associated with longer gestational length (adjusted beta coefficient [aβ] = 0.36, 95% confidence interval [CI] = 0.03, 0.68) and higher birth weight (aβ = 124.71, 95% CI = 35.75, 213.66). A higher prenatal positive affect was associated with longer gestational length (aβ = 0.38, 95% CI = 0.06, 0.70), whereas higher levels of negative affect and depression symptoms were associated with smaller birth weight and shorter gestational length, respectively. We found no association between life evaluation, anxiety, and birth outcomes.

**Table 2.** Effects of prenatal positive and negative mental health on birth outcomes: estimates from linear regression models.

	Gestational age		Birth weight	
Variable	cβ (95% CI)	aβ (95% CI)	cβ (95% CI)	aβ (95% CI)
Life evaluation	−0.11 (−0.21, 0.41)	0.17 (−0.16, 0.50)	101.16 (15.32, 187.01)*	87.84 (−1.72, 177.39)
Eudaimonic well-being	0.31 (−0.01, 0.62)	0.36 (0.03, 0.68)*	129.80 (44.65, 214.95)**	124.71 (35.75, 213.66)**
Positive affect	0.44 (0.13, 0.75)**	0.38 (0.06, 0.70)*	100.31 (12.86, 187.77)*	59.25 (−27.65, 146.16)
Negative affect	−0.27 (−0.77, 0.04)	−0.23 (−0.54, 0.09)	−105.19 (−190.71, −19.67)*	−93.51 (−178.35, −8.67)*
Depression	−0.11 (−0.28, 0.06)	−0.37 (−0.62, −0.11)**	−26.97 (−75.38, 21.43)	−66.99 (−138.18, 4.18)
Anxiety	−0.08 (−0.24, 0.09)	−0.17 (−0.44, 0.09)	−19.21 (−68.05, 29.63)	−55.42 (−127.23, 16.39)

*Note*: Results were adjusted for maternal age, employment status, family income, unplanned pregnancy, smoking status, maternal perceived health status, baby sex, and parity.

*p < 0.05, **p < 0.01.

*Abbreviations:* aβ, adjusted beta coefficient; cβ, crude beta coefficient; CI, confidence interval.

Table 3 presents the longitudinal effects of maternal mental health on child development during the first 2 years of life. Using GEE models, after adjustment for potential confounders, higher prenatal eudaimonic well-being was significantly associated with lower risks of suspected developmental delay, particularly among children of multiparous mothers (adjusted odds ratio [aOR] = 0.18, 95% CI = 0.05, 0.70). Higher levels of prenatal depression and anxiety were associated with increased risks of suspected developmental delay in children of primiparous mothers. We observed no associations between life evaluation, positive and negative affect, and suspected child developmental delay.

**Table 3.** Effects of maternal positive and negative mental health from prenatal to postpartum periods on child development during the first 2 years based on parity: estimates from generalized estimating equation models.

	Total sample		Primiparous		Multiparous	
Variable	cOR (95% CI)	aOR (95% CI)	cOR (95% CI)	aOR (95% CI)	cOR (95% CI)	aOR (95% CI)
Life evaluation						
Prenatal						
Lower	1	1	1	1	1	1
Higher	0.71 (0.43, 1.18)	0.73 (0.43, 1.26)	0.80 (0.42, 1.50)	0.83 (0.42, 1.64)	0.58 (0.25, 1.38)	0.65 (0.23, 1.80)
Postpartum						
Lower	1	1	1	1	1	1
Higher	0.88 (0.56, 1.37)	0.95 (0.58, 1.54)	0.90 (0.53, 1.53)	1.02 (0.57, 1.87)	0.85 (0.38, 1.90)	0.80 (0.33, 1.93)
Eudaimonic well-being						
Prenatal						
Lower	1	1	1	1	1	1
Higher	0.55 (0.32, 0.95)*	0.58 (0.32, 1.04)	0.83 (0.42, 1.66)	0.93 (0.45, 1.93)	0.23 (0.09, 0.59)**	0.18 (0.05, 0.70)*
Postpartum						
Lower	1	1	1	1	1	1
Higher	1.19 (0.73, 1.96)	1.26 (0.76, 2.09)	1.04 (0.57, 1.90)	1.11 (0.59, 2.06)	1.87 (0.79, 4.42)	2.06 (0.80, 5.35)
Positive affect						
Prenatal						
Lower	1	1	1	1	1	1
Higher	0.67 (0.41, 1.08)	0.76 (0.46, 1.26)	0.65 (0.7, 1.15)	0.70 (0.40, 1.23)	0.71 (0.29, 1.72)	0.94 (0.38, 2.35)
Postpartum						
Lower	1	1	1	1	1	1
Higher	1.13 (0.69, 1.85)	1.09 (0.65, 1.81)	1.18 (0.66, 2.11)	1.30 (0.72, 2.34)	1.06 (0.42, 2.67)	0.79 (0.31, 2.04)
Negative affect						
Prenatal						
Lower	1	1	1	1	1	1
Higher	1.49 (0.86, 2.56)	1.38 (0.77, 2.47)	1.44 (0.76, 2.72)	1.42 (0.70, 2.85)	1.50 (055, 4.10)	1.16 (0.36, 3.74)
Postpartum						
Lower	1	1	1	1	1	1
Higher	0.96 (0.59, 1.55)	1.07 (0.66, 1.74)	0.78 (0.44, 1.41)	0.79 (0.44, 1.45)	1.37 (0.61, 3.09)	2.17 (1.01, 4.67)^┼^
Depression						
Prenatal						
Lower	1	1	1	1	1	1
Higher	1.60 (1.21, 2.12)**	1.6191.21, 2.15)**	1.85 (1.29, 2.66)**	1.72 (1.20, 2.48)**	1.26 (0.81, 1.97)	1.36 (0.86, 2.16)
Postpartum						
Lower	1	1	1	1	1	1
Higher	1.31 (0.89, 1.92)	1.35 (0.91, 2.01)	1.13 (0.69, 1.86)	1.21 (0.73, 2.01)	1.65 (0.91, 2.98)	1.74 (0.90, 3.35)
Anxiety						
Prenatal						
Lower	1	1	1	1	1	1
Higher	1.55 (1.17, 2.05)**	1.58 (1.19, 2.11)**	1.93 (1.35, 2.74)***	1.79 (1.24, 2.56)**	1.08 (0.68, 1.72)	1.16 (0.71, 1.87)
Postpartum						
Lower	1	1	1	1	1	1
Higher	1.11 (0.81, 1.51)	1.15 (0.83, 1.58)	1.00 (0.67, 1.48)	1.04 (0.69, 1.56)	1.24 (0.74, 2.08)	1.30 (0.76, 2.24)

*Note*: Results were adjusted for maternal age, education, family income, unplanned pregnancy, baby sex, and baby health status.

*p < 0.05, **p < 0.01, ***p < 0.001, ^┼^*p* = 0.05.

## Discussion

To the best of our knowledge, this is the first prospective study to broadly examine the impact of maternal SWB on birth outcomes and child early development from early pregnancy to 2 years postpartum while considering the effects of parity. We observed that prenatal eudaimonic well-being was associated with longer gestational length and larger birth weight, and positive affect was associated with longer gestational length. We further observed that prenatal eudaimonic well-being was independently associated with an 82% decreased risk of suspected developmental delay up to 2 years of age among children of multiparous mothers. In addition, higher levels of prenatal depression and anxiety were associated with increased risks of suspected developmental delay among children of primiparous mothers. However, life evaluation and positive and negative affect had no significant association.

In this study, positive prenatal mental health (i.e., eudaimonic well-being and positive affect) was associated with better birth outcomes, and higher negative affect and depression were associated with worse birth outcomes. Previous studies have focused mainly on the effects of maternal mental illnesses and have reported consistent results. Antepartum depression and anxiety have been reported to be significantly associated with preterm birth and low birth weight [31, 32]. Furthermore, limited evidence suggests that higher prenatal eudaimonic well-being and positive affect are associated with lower risks of small gestational weeks and birth weight. A study reported a positive association between increasing positive maternal affect throughout pregnancy and birth outcomes [33]. Another study identified that positive appraisal was associated with less distress and high coping stability throughout pregnancy [34]. However, this study observed a null finding for life evaluation, perhaps due to researchers typically equating life evaluation with overall life satisfaction. Moreover, this study did not observe any association between anxiety and birth outcomes. Anxiety has been linked to premature birth and low birth weight [35]. The inconsistency and null findings may be explained by variability in assessment tools and differences in sample characteristics (e.g., higher socioeconomic status with better resources and social support) [36, 37].

Researchers have identified a negative association between maternal depression and anxiety and child cognitive development at 2 and 3 years [38]. Other studies have also revealed a negative association between antenatal depression and child self-regulation [39] and development [40, 41]. Consistent with the findings of these studies, we found that higher levels of prenatal depression and anxiety were associated with elevated risks of child suspected developmental delay in children of primiparous mothers. Moreover, in our study, we observed beneficial effects of antenatal eudaimonic well-being on child developmental outcomes, especially among children of multiparous mothers. Consistent with our findings, Clayborne et al. (2022) identified that positive maternal mental health during pregnancy was associated with positive developmental outcomes in children aged 5 [16].

Maternal mental health during pregnancy has been suggested to exert effects on child psychopathology and subsequent development, irrespective of maternal postnatal mental health. Specifically, through the biological pathway of the effect of maternal emotions on birth outcomes, increased levels of maternal cortisol and placental corticotropin-releasing hormone CRH during pregnancy were further observed to be associated with reduced fetal growth, possibly affecting subsequent development in children [42, 43]. In addition, optimistic women with a higher SWB were more proactive and motivated to engage in healthy behaviors (e.g., physical activity, a well-balanced, and nutritious diet) [44, 45], consequently promoting healthy fetal growth and leading to positive developmental outcomes for the offspring [46]. A mediating analysis further revealed that higher maternal SWB positively influenced child cognitive and linguistic development through more adaptive parent–child interaction patterns [17]. The effects of parity with the various demands and challenges faced by first- and second-time mothers are noteworthy. Although the reasons underlying the beneficial effects of the SWB of pregnant women, particularly multiparous women, on child development remain unclear, a higher socioeconomic status with better family support and resources for those who wish to have two or more children may be key factors, as Taiwan’s total fertility rate was estimated to be the lowest in the world in 2021. Further studies are needed to elucidate these findings.

In the postpartum period, maternal mental health has been linked to child developmental outcomes through parental behavior [14, 47, 48]. However, we did not observe a significant association between postpartum positive and negative mental health and child development. Because maternal postnatal emotions are often carried over from the prenatal period [49], independent effects in the postpartum period may be reduced. Null and inconsistent findings may further be explained by the moderating effect of parity, assessment methods, and cultural context applied.

Regarding mood changes during the perinatal period, a previous study found that mood levels are increased around the time of birth and subsequently return to baseline in less than 2 years [50]. In our study, scores of maternal eudaimonic well-being significantly decreased from early pregnancy to 2 years postpartum, whereas positive affect scores significantly decreased from T3 to T4 and then returned to the initial level at 6 months postpartum. This may possibly reflect the circumstances that depression levels may increase in the early postpartum period as a result of substantial changes accompanied by increased stress [51, 52]. The reasons for and effects of the lack of increase in maternal happiness around childbirth observed in this study must be further investigated.

This study has valuable implications. It described the trends in eudaimonic well-being and positive affect scores from early pregnancy to 2 years postpartum according to parity and identified the specific period in which intervention was required to improve well-being. First, maternal eudaimonic well-being and positive affect declined after birth, emphasizing the importance of family support during that time. Second, because well-being is modifiable [53, 54], interventions for ensuring positive well-being in prenatal care settings are necessary [55], with particular emphasis on experienced mothers. Finally, because depression and anxiety during pregnancy were associated with suspected developmental delay among children of inexperienced mothers, interventions aimed at reducing prenatal mental illness, such as stress coping and emotion management, are crucial for inexperienced mothers.

Our study has several strengths. First, we used a prospective follow-up research design, which allowed us to examine longitudinal changes in women’s SWB scores in multiple domains from early pregnancy to 2 years postpartum, with reduced recall bias. Second, we assessed the effects of maternal SWB in the first 2 years of child’s life, which is the period of remarkable early brain growth and child development. Third, evaluations conducted at seven time points enabled a trimester-specific approach and the identification of relevant time-varying factors for modeling to appropriately consider the timing of important factors and to adjust for major confounders. Fourth, the assessment of the potential modifier effect of parity facilitates the identification of higher risk groups. Finally, our overall follow-up rate was approximately 60% [55], which is considered appropriate for a cohort study [56]. No significant differences in demographics (e.g., age and education level) were observed between women who were lost to follow-up and those who remained in the study.

Our study also has some limitations. First, we recruited participants from medical centers in metropolitan Taipei, including older women with a relatively high socioeconomic status. Our findings are likely to be understated because couples with poorer marital relationships or lower levels of SWB may be more reluctant to participate or more likely to be lost to follow-up. Second, because our surveys were based on self-reporting questionnaires, potential bias due to social desirability and perception bias cannot be ruled out. Furthermore, the Taipei II was used to screen for suspected developmental delays among children instead of clinical diagnostics. Finally, the possibility of reverse causality cannot be ruled out, as prenatal emotions may be generated as maternal responses and perception of potential medical risks on child developmental outcomes.

## Conclusion

This study examined how fundamental components of positive maternal mental health and maternal mental illness affect birth outcomes and child development from early pregnancy to 2 years postpartum. Our results demonstrated that prenatal eudaimonic well-being positively affects birth outcomes and child development, especially in children of multiparous mothers. Longitudinal study with a follow-up period of more than 2 years is required to determine the consistency of the association between eudaimonic well-being and child development by parity, including physiological factors.

## Data Availability

A request to gain access to the data can be made by contacting the corresponding author. Access can be granted subject to the Institutional Review Board (IRB) and the research collaborative agreement guidelines. This is a requirement mandated for this research study by our ethics committee and funders.

## Abbreviations

AOR: adjusted odds ratio
CI: confidence interval
COR: crude odds ratio

## Supplementary material

For supplementary material accompanying this paper visit https://doi.org/10.1192/j.eurpsy.2022.2338. click here to view supplementary material
