# Supplementary material for: Effects of prenatal subjective well-being on birth outcomes and child development: A longitudinal study
Source: Eur Psychiatry. 2022 Nov 4;65(1):e77. doi: 10.1192/j.eurpsy.2022.2338 (PMC9706305; doi:10.1192/j.eurpsy.2022.2338)
Supplement: Supplementary file 1 [file S0924933822023380sup001.docx]

S. Table 1. Distribution of prenatal life positive and negative affect by demographics and maternal and infant characteristics

| Variables | Positive affect |  |  | Negative affect |  |  |
| --- | --- | --- | --- | --- | --- | --- |
|  | Lower | Higher | P_value | Lower | Higher | P_value |
|  | n (%) | n (%) |  | n (%) | n (%) |  |
| **Maternal age** |  |  |  |  |  |  |
| <35 | 93(33.8) | 182(66.2) |  | 193(70.2) | 82(29.8) |  |
| ≥35 | 59(33.7) | 116(66.3) | 0.98 | 140(80.0) | 35(20.0) | 0.02* |
| **Education level** |  |  |  |  |  |  |
| High school or below | 6(25.0) | 18(75.0) |  | 19(79.2) | 5(20.8) |  |
| undergraduate and above | 147(34.3) | 281(65.7) | 0.35 | 313(73.1) | 115(26.9) | 0.52 |
| **Employment status** |  |  |  |  |  |  |
| yes | 127(32.9) | 259(67.1) |  | 292(75.7) | 94(24.3) |  |
| No | 25(37.9) | 41(62.1) | 0.43 | 41(62.1) | 25(37.9) | 0.02* |
| **Monthly family income** |  |  |  |  |  |  |
| Less than NT$ 60,000 | 35(48.0) | 38(52.0) |  | 46(63.0) | 27(37.0) |  |
| NT$ 60,000-NT$ 100,000 | 73(33.5) | 145(66.5) | 0.008** | 156(71.6) | 62(28.4) | 0.007** |
| More than NT$ 100,000 | 44(27.2) | 118(72.8) |  | 132(81.5) | 30(18.5) |  |
| **Smoking** |  |  |  |  |  |  |
| Yes | 4(36.4) | 7(63.6) | 0.87 | 6(54.6) | 5(45.4) | 0.15 |
| No | 147(34.1) | 284(65.9) |  | 318(73.8) | 113(26.2) |  |
| **Drinking** |  |  |  |  |  |  |
| yes | 6(35.3) | 11(64.7) |  | 11(64.7) | 6(35.3) |  |
| No | 146(33.6) | 289(66.4) | 0.88 | 322(74.0) | 113(26.0) | 0.39 |
| **Mother main care giver** |  |  |  |  |  |  |
| yes | 136(33.8) | 267(66.2) |  | 300(74.4) | 103(25.6) |  |
| No | 17(34.7) | 32(65.3) | 0.89 | 33(67.3) | 16(32.7) | 0.29 |
| **Depression** |  |  |  |  |  |  |
| Higher | 98(63.6) | 56(36.4) |  | 63(40.9) | 91(59.1) |  |
| Lower | 49(17.2) | 236(82.8) | <.001*** | 260(91.2) | 25(8.8) | <.001*** |
| **Anxiety** |  |  |  |  |  |  |
| Higher | 84(68.3) | 39(31.7) |  | 49(39.8) | 74(60.2) |  |
| Lower | 63(19.9) | 253(80.1) | <.001*** | 274(86.7) | 42(13.3) | <.001*** |
| **Parity** |  |  |  |  |  |  |
| Primiparous | 98(32.6) | 203(67.4) |  | 218(72.4) | 83(27.6) |  |
| Multiparous | 55(36.0) | 98(64.0) | 0.47 | 116(75.8) | 37(24.2) | 0.45 |
| **Mother perceived health** |  |  |  |  |  |  |
| Very Good | 2(5.3) | 36(94.7) |  | 34(89.5) | 4(10.5) |  |
| Good | 138(36.3) | 242(63.7) | <.001*** | 316(83.1) | 64(16.9) | <.001*** |
| Bad | 27(77.1) | 8(22.9) |  | 22(63.2) | 13(36.8) |  |
| **Fetus health problem** |  |  |  |  |  |  |
| yes | 69(36.0) | 126(64.0) |  | 142(74.0) | 50(26.0) |  |
| No | 86(32.8) | 175(67.2) | 0.65 | 192(73.4) | 69(26.6) | 0.93 |
| **Infant's sex** |  |  |  |  |  |  |
| Boy | 70(31.2) | 154(68.8) |  | 174(77.7) | 50(22.3) |  |
| Girl | 79(35.9) | 141(64.1) | 0.30 | 154(70.0) | 66(30.0) | 0.52 |
| **Planned pregnancy** |  |  |  |  |  |  |
| Yes | 49(36.0) | 87(64.0) |  | 99(72.8) | 37(27.2) |  |
| No | 101(32.2) | 213(67.8) | 0.43 | 233(74.2) | 81(25.8) | 0.76 |

*p < 0.05, **p < 0.01, ***p < 0.001

The total count for each variable may vary because of missing values.

S. Table 2. Distribution of prenatal depression and anxiety affect by demographics and maternal and infant characteristics

| Variables | Depression |  |  | Anxiety |  |  |
| --- | --- | --- | --- | --- | --- | --- |
|  | Lower | Higher | P_value | Lower | Higher | P_value |
|  | n (%) | n (%) |  | n (%) | n (%) |  |
| **Maternal age** |  |  |  |  |  |  |
| <35 | 528(66.8) | 262(33.2) |  | 561(71.0) | 229(29.0) |  |
| ≥35 | 307(70.7) | 127(29.3) | 0.12 | 295(68.0) | 139(32.0) | 0.34 |
| **Education level** |  |  |  |  |  |  |
| High school or below | 43(55.8) | 34(42.2) |  | 50(64.9) | 27(35.1) |  |
| undergraduate and above | 794(69.1) | 355(30.9) | 0.01* | 805(70.0) | 344(30.0) | 0.40 |
| **Employment status** |  |  |  |  |  |  |
| yes | 730(69.8) | 315(30.2) |  | 738(70.6) | 307(29.4) |  |
| No | 110(61.4) | 69(38.6) | 0.03* | 122(68.2) | 57(31.8) | 0.56 |
| **Monthly family income** |  |  |  |  |  |  |
| Less than NT$ 60,000 | 143(58.0) | 104(42.0) |  | 155(62.7) | 92(37.3) |  |
| NT$ 60,000-NT$ 100,000 | 386(68.1) | 184(31.9) | <0.001*** | 398(69.8) | 172(30.2) | 0.003** |
| More than NT$ 100,000 | 306(75.6) | 99(24.4) |  | 304(75.1) | 101(24.9) |  |
| **Smoking** |  |  |  |  |  |  |
| Yes | 20(52.6) | 18(47.4) | 0.02* | 25(65.8) | 13(34.2) | 0.49 |
| No | 797(68.9) | 360(31.1) |  | 808(70.0) | 348(30.0) |  |
| **Drinking** |  |  |  |  |  |  |
| yes | 25(64.1) | 14(35.9) |  | 26(66.8) | 13(33.3) |  |
| No | 805(68.6) | 369(31.4) | 0.54 | 826(70.4) | 348(29.6) | 0.61 |
| **Mother main care giver** |  |  |  |  |  |  |
| yes | 453(67.5) | 218(32.5) |  | 480(71.5) | 191(28.5) |  |
| No | 66(68.0) | 31(32.0) | 0.89 | 65(67.0) | 32(33.0) | 0.27 |
| **Parity** |  |  |  |  |  |  |
| Primiparous | 527(68.7) | 240(31.1) |  | 548(71.4) | 219(28.6) |  |
| Multiparous | 309(67.8) | 147(32.2) | 0.77 | 306(67.1) | 150(32.9) | 0.15 |
| **Mother perceived health** |  |  |  |  |  |  |
| Very Good | 167(79.1) | 44(20.9) |  | 177(83.9) | 34(16.1) |  |
| Good | 315(61.4) | 198(38.6) | <.001*** | 327(63.7) | 186(36.6) | <.001*** |
| Bad | 38(84.4) | 7(15.6) |  | 41(91.1) | 4(8.9) |  |
| **Fetus health problem** |  |  |  |  |  |  |
| yes | 12(60.) | 8(40.0) |  | 14(70.0) | 6(30.0) |  |
| No | 505(68.3) | 239(31.7) | 0.63 | 525(71.0) | 214(29.0) | 0.86 |
| **Infant's sex** |  |  |  |  |  |  |
| Boy | 437(70.1) | 186(29.0) |  | 439(70.5) | 184(29.5) |  |
| Girl | 395(68.0) | 186(32.0) | 0.52 | 399(68.7) | 182(31.3) | 0.54 |
| **Planned pregnancy** |  |  |  |  |  |  |
| Yes | 242(63.5) | 139(36.5) |  | 243(63.8) | 138(36.2) |  |
| No | 586(70.0) | 251(30.0) | 0.03* | 609(72.6) | 228(27.4) | 0.001** |

*p < 0.05, **p < 0.01, ***p < 0.001

The total count for each variable may vary because of missing values.
